# Case report: A case of abrupt stroke as the first symptom of neurobrucellosis

**DOI:** 10.3389/fneur.2023.1066042

**Published:** 2023-02-22

**Authors:** Ying Liu, Yan Gu

**Affiliations:** Department of Radiology, The Affiliated Lianyungang Hospital of Xuzhou Medical University, Lianyungang, Jiangsu, China

**Keywords:** intracranial infection, Bacterium burgeri, cerebral infarction, vasculitis, stroke

## Abstract

Acute cerebral infarction caused by small artery inflammatory disease, which is triggered by neurobrucellosis, is a rare condition that can be easily misdiagnosed. Neurobrucellosis is a rare complication of brucellosis that can present clinically as meningitis, meningoencephalitis, myelitis, neuritis, or psychosis. In this study, we report the case of a patient with neurobrucellosis who was hospitalized in the First People's Hospital of Lianyungang in September 2022; the primary symptom was weakness in the left limb for 14 h. The patient was discharged after receiving symptomatic and anti-Brucella medication.

## Background

Contact with diseased animals or non-pasteurized dairy products is one of the primary mechanisms of Brucella transmission from animals to humans; this is one of the most prevalent zoonotic infections globally (1). The imaging symptoms of neurobrucella infection, an uncommon consequence of systemic brucellosis, can be categorized into four categories: normal, inflammation (abnormal enhancement), white matter changes, and vascular changes (2). In this study, we describe the diagnosis and course of the patient with neurobrucellosis who was treated at Lianyungang First People's Hospital in September 2022. We discuss our experience in relation to the existing literature to facilitate radiologists when diagnosing neurobrucellosis.

## Case information

The patient was a 44-year-old man, 175 cm in height, and 78 kg in weight. His main complaint upon admission to the hospital was left-sided limb weakness for 14 h. When using the restroom 14 h earlier, the patient discovered the left-sided limb weakness but was still able to stand and walk. He was unable to hold things in his hands but had no slurred speech, no choking, or coughing, no difficulty in swallowing, no impairment of consciousness or limb convulsions, and no urinary or fecal incontinence. The patient went to the county hospital soon after the condition began and was diagnosed with cerebral infarction and cervical spondylosis. However, there was no specific diagnosis or therapeutic option. The patient felt that the left-sided limb weakness had become aggravated and was advised to attend a higher level hospital for medical treatment. He was subsequently admitted to the Lianyungang First People's Hospital on 23 August 2022 for emergency treatment and received consultation from the Neuro-interventional Department. There was no indication for surgery, and he was admitted for further diagnosis and treatment for suspected cerebral infarction.

The physical examination showed a body temperature of 36.6°C, a heart rate of 60 beats/min, a respiration rate of 18 breaths/min, and a blood pressure of 126/75 mmHg. Heart, lung, and abdominal examinations were negative. Consciousness was clear, the left upper limb muscle strength grade was three, the left lower limb muscle strength grade was four, and the right limb muscle strength grade was five. He had normal muscle tone, left finger nose inability, heel and knee tibial stability, and a reduced left limb pinprick sensation. Tendon reflexes were evident on both sides, there was no ankle or bin clonus, and the left Bartholomew's sign was positive. The neck was soft with a negative Gram's sign and Brønsted's sign. The National Institute of Health stroke scale (NIHSS) score was seven and the modified Rankin Scale (MRS) score was three. Upon admission, the TOAST classification suggested a diagnosis of unexplained cerebral infarction.

### Auxiliary examination, diagnosis, and therapeutic actions

The patient complained of left limb weakness, fever, headache, and general sleep disorder. The patient was given a clinical diagnosis of cerebral infarction after consulting with the Department of Neuro-interventional Surgery; consultation determined that this case did not match the criteria for thrombolysis. After receiving symptomatic treatments, including antiplatelet medication, cholesterol regulation, and circulation improvement, the patient still experienced recurring fever.

**Figure 1:**
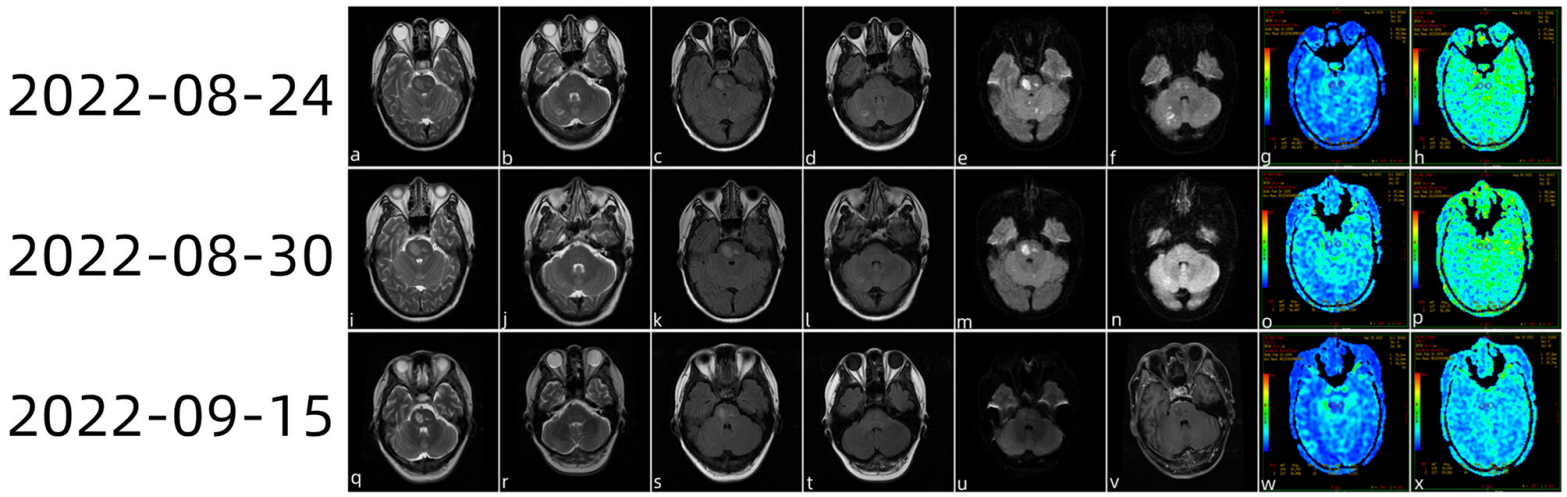
Head magnetic resonance imaging (MRI) plain scan 3.0 examination diagnosis (a–f) on 24 August 2022 showing multiple acute foci in the brainstem and bilateral cerebellar hemispheres. ASL (g, h) showed a mild reduction in the perfusion of the lesions. On 30 August 2022, the cranial MRI (i–n) showed that the bilateral cerebellar acute infarction had improved and that the brainstem acute infarction had reduced slightly. ASL revealed slight hypoperfusion when PLD (o) was 1.5 s and the cerebral stem perfusion was similar to that of the contralateral side at 2.5 s (p). On 15 September 2022, the patient came to the hospital for review (q–x); brain stem lesions were smaller than those of half a month ago, while magnetic resonance enhancement of the brain showed (v) heterogeneous enhanced foci in the brain stem.

On 24 August 2022 (Figure 1), multiple acute foci in the brainstem and contralateral cerebellar hemispheres were identified by cranial magnetic resonance imaging (MRI) plain 3.0 scanning. Arterial spin labeling (ASL) revealed modest hypoperfusion of the lesions, and cranial magnetic resonance angiography (MRA) did not identify the appreciable macrovascular abnormalities.

A routine blood count (on 26 August 2022) showed a neutrophil ratio of 81.5% (reference range: 40–75%), a lymphocyte percentage of 11.8% (reference range: 20–50%), and an absolute lymphocyte value of 0.90 × 10^9^/L (1.8–6.3 × 10^9^/L). The TORCH eight items (evaluated on 27 August 2022) were as follows: a cytomegalovirus IgG antibody concentration >500.00 U/ml (0–0.49, negative), a rubella virus IgG antibody concentration of 85.45 IU/ml (< 10 negative), and a herpes simplex virus type I IgG concentration of 14.350 (+) COI+ (0–0.59 negative). The following did not show any abnormalities: tuberculosis infection *T*-cell test, fecal occult blood test, routine urinary tests, emergency kidney function, stool tests, blood bacterial culture (adult), and drug sensitivity assays. Anti-β2-glycoprotein 1 antibody assays (enzyme immunoassay), smears for novel cryptococci, bacterial smears, smears for acid-fast bacilli (mycobacterium tuberculosis), blood bacterial cultures (adult) and drug sensitivity acids, quantitative EBV nucleic acid assays (EBDNAs), cytomegalovirus nucleic acid assays (CMVDNAs), G tests, and the GM test did not reveal any significant abnormalities.

The patient still experienced intermittent fever; a history of herding sheep was revealed by the follow-up medical history. As a consequence, we carried out further tests on the cerebrospinal fluid, including the routine cerebrospinal fluid examination (29 August 2022), Pan's reaction (+), and cerebrospinal fluid biochemistry (29 August 2022). White blood cell count was 150 × 10∧6 (range: < 10 × 10∧6). The lymphocyte ratio was 31% (range: < 20%), chlorine was 107.6 mmol/L (range: 110–130 mmol/L), cerebrospinal fluid protein was 405.70 mg/dl (range: 12–60 mg/dl), and cerebrospinal fluid IGG was 327 mg/L (range: 10–40 mg/L). Combined with the patient's medical history and positivity for Brucella antibody in the serum, the patient was clinically diagnosed with Brucella encephalitis and cerebral infarction. On 30 August 2022, cranial magnetic resonance imaging showed that the bilateral cerebellar acute infarction was better than before and that the acute infarction in the brainstem had reduced slightly. ASL revealed slight hypoperfusion when post-label delay (PLD) was 1.5 s, while cerebral stem perfusion was similar to that of the contralateral side at 2.5 s. The patient was released from the hospital following anti-infection and anti-platelet medication, cholesterol control, circulation improvement, headache relief, and anti-viral treatment. The patient re-visited the hospital on 15 September 2022 for a review; cranial magnetic resonance enhancement revealed heterogeneous enhancement foci in the brainstem.

## Discussion

Brucella are aerobic Gram-negative bacilli that reside mainly in the cells of domestic or wild animals (3). Brucellosis is difficult to diagnose early due to the lack of specific signs or symptoms of infection. Neurobrucellosis is a rare complication and can manifest clinically as meningitis, meningoencephalitis, myelitis, neuritis, or psychosis (4). Cerebral infarction caused by cerebrovascular disease as the first clinical manifestation is rare and can therefore be misdiagnosed easily. *Brinell coli* bacteria can invade due to direct damage of nerve tissue caused by endotoxins or bacteria or during immune inflammation caused by indirect damage caused by the central nervous system, and several nervous system symptoms, such as visual papillary edema, seizures, and confusion (5). In addition, patients may also experience non-specific symptoms, such as fever, headache, fatigue, and weight loss.

A yearly incidence of 500,000 cases of brucellosis is estimated by the World Health Organization (6). Despite the fact that it occurs globally, it is commonly misdiagnosis and unreported. Neurobrucellosis is a rare complication of brucellosis, which affects 3–10% of those with brucellosis (7). Despite the low mortality rate, neurological sequelae after neurobrucellosis are still common. It is estimated that 20–30% of patients with neurobrucellosis develop neurological sequelae (8).

In a previous study, Ceran et al. (2) reported some unusual clinical findings in the cases of neurobrucellosis and identified vasculitis of the left middle cerebral artery in one out of 18 confirmed cases; this resulted in an acute infarction of the feeding area of this vessel. In another study, Peizhe et al. reported a case of neurobrucellosis in a young man presenting with thalamic apoplexy (3); this patient suffered acute onset. Cranial MRI examination revealed acute infarction in the left thalamus although there were no obvious abnormalities in the intracranial arteries. Considering that the acute lesion of the left thalamus was not consistent with the distribution area of the thalamic feeding artery, the patient was diagnosed with cerebral vascular disease with clinical manifestations of intracranial venous system thrombosis. In our case, the patient had multiple acute infarcts in the brainstem and bilateral cerebellar hemispheres. These infarcts were in line with the perforator blood supply distribution area of the bridging vessels and vertebral arteries, which is most likely a kind of small artery inflammatory disease that may be triggered by Brucella. The brain stem enhancement was most likely due to damage being incurred by the local blood–brain barrier after cerebral infarction, thus resulting in the leakage of contrast media.

In addition, we need to rule out the possibility of perforating artery disease (PAD) such as atherosclerosis for small vessels causing the perforator artery infarction. Considering that the patient was young (44 years old) and had no risk factors such as hypertension, hyperlipidemia, diabetes, and other risk factors of atherosclerosis, intracranial MRA showed that the blood vessels of the anterior and posterior circulation were normally distributed, the blood vessel walls were smooth, pontine artery and vertebral artery were not significantly plaque formation, and lumen was not significantly stenosis, and we believed that the possibility of atherosclerosis was not very high.

The diagnostic criteria (9) for the diagnosis of neurobrucellosis are as follows: (1) clinical features of the illness compatible with a known neurobrucellosis syndrome; (2) typical CSF changes (pleocytosis, elevated protein concentration); (3) positive results of either blood or bone marrow or CSF culture or appropriate serological tests (e.g., agglutination test titers of >1:160 in blood or any positive titer in CSF); (4) clinical improvement after starting an appropriate treatment; and (5) inability to prove a more suitable alternative diagnosis. The patient presented with acute stroke in his youth as the first presentation without other high-risk factors of cerebrovascular disease. Combined with the history of herding sheep, the positive result of Brucella antibody in the serum, cerebrospinal fluid changes, and the clinical symptoms improved after anti-Brucella treatment; this was consistent with the diagnosis of neurobrucellosis.

Neurobrucellosis is a multisystem disease with a broad spectrum of clinical manifestations (5). The acute stage can be manifested as chills, fever, sweating, etc.; the disease progresses to the chronic phase after 1 year or more, which is divided into chronic activity type and chronic stable type. Presenting a severe and persistent headache is one of the most significant hallmarks of neurobrucellosis (10). Moreover, blurred vision, loss of hearing, and confusion were also found to be common among patients with neurobrucellosis. Meningitis is the most typical symptom of central nervous system involvement, and meningeal irritation is the most common symptom (11). Additionally mentioned meningovascular side effects include subarachnoid hemorrhages, ischemic strokes, and mycotic aneurysms. Patients can also present with cranial nerve involvement, non-central facial paralysis, and sensorineural hearing loss with the vestibulocochlear nerve involved, which is the most commonly involved central nervous (12). Other neurologic manifestations (13, 14), such as isolated intracranial hypertension, psychiatric symptoms, and Guillain–Barre syndrome, are rare.

Antibiotic choice, dosage, and duration of antibacterial therapy for neurobrucellosis are still controversial, and a combination of three or four antibiotics is usually used until the clinical manifestations resolve and the cerebrospinal fluid returns to normal. Ceftriaxone combined with rifampicin and doxycycline was selected as the antibiotic treatment plan for this patient. After 2 weeks, the patient's symptoms were significantly relieved and cerebrospinal fluid was re-examined as usual. Due to the possibility of recurrence, we recommend that patients be followed up every 3 months. The prognosis of neurobrucella varies with the clinical manifestations. Ceran et al. (2) concluded that patients presenting with meningitis usually have a good prognosis, while patients involving brain parenchyma and spinal cord often have serious sequelae.

The specific pathogenesis of transient ischemic attack or ischemic stroke caused by Brucella remains unclear; there are only a few reports of its invasion into cerebral blood vessels. There are several possible mechanisms that might be responsible for these effects (2). First, the invasion of the endocardium by Brucella may lead to the formation of vegetative organisms; the shedding of these organisms might cause infarction of the intracranial artery. Second, the inflammatory immune response caused by Brucella infection or its endotoxins may cause small vasculitis, vasospasm, or inflammation of the venous system, thus resulting in infarction, micro-bleeding, or venous thrombosis of the arterial feeding area.

Neuroimaging examinations are of great significance in the diagnosis of neurobrucellosis but must be combined with the clinical history of patients to have diagnostic value. Radiologists need to improve their understanding of neurobrucellosis. When patients with recurrent fever and unexplained stroke manifestations cannot be explained by simple cerebrovascular lesions, an enhanced understanding of the patient's history is needed to assist the clinicians in reaching a timely diagnosis.

## Conclusion

Neurobrucellosis lacks specific neuroimaging findings and clinical signs. The diagnosis of this condition depends on the specific symptoms of systemic Brucellosis infection. If neuroimaging changes exist and cannot be reasonably explained by other neurological diseases, it is important to consider the possibility of neurobrucellosis.

## Data availability statement

The original contributions presented in the study are included in the article/supplementary material, further inquiries can be directed to the corresponding author.

## Ethics statement

Written informed consent was obtained from the individual(s) for the publication of any potentially identifiable images or data included in this article.

## Author contributions

YL: writing the original draft. YG: writing, reviewing, editing, and funding acquisition. All authors agreed to be accountable for the content of the study.
